# Villous Architecture in Colorectal Adenomas: Molecular Pathogenesis, Epithelial Plasticity, and Malignant Risk

**DOI:** 10.3390/cells15181685

**Published:** 2026-09-17

**Authors:** Zedong Hu, Tristan Vanoverbeke, Ke Yin, Alexander Jans, Chuanmei Carlotta Arpaia, Raf Bisschops, Paméla Baldin, Sabine Tejpar, Hubert Piessevaux

**Affiliations:** 1 Digestive Oncology, Department of Oncology, KU Leuven, 3000 Leuven, Belgium; 2 Department of Gastroenterology and Hepatology, UZ Leuven, 3000 Leuven, Belgium; 3 Department of Translational Research in Gastrointestinal Diseases (TARGID), KU Leuven, 3000 Leuven, Belgium; 4 Department of Hepato-Gastroenterology, Cliniques Universitaires Saint-Luc, Université Catholique de Louvain, 1200 Brussels, Belgium; 5 Department of Pathology, Cliniques Universitaires Saint-Luc, Université Catholique de Louvain, 1200 Brussels, Belgium

**Keywords:** colorectal adenoma, villous architecture, epithelial plasticity, YAP, colorectal carcinogenesis

## Abstract

Villous morphology is routinely used to stratify risk in conventional colorectal adenomas, yet it remains largely interpreted as a visual histological descriptor. This review proposes a different view: villous architecture is a graded, spatially organized epithelial state that links premalignant tissue remodeling to malignant potential. Across histological, molecular, and microenvironmental studies, villous-containing adenomas show convergent features of WNT-driven stem-like expansion, YAP-associated plasticity, fetal-regenerative or fetal-metaplastic transcriptional programs, mucin remodeling, stromal activation, immune attenuation, and genomic instability. These features appear to arise through interacting routes rather than through a single deterministic mutation. This framework helps explain why villous proportion predicts risk imperfectly: it captures a composite biological state but does not resolve its individual components. Quantitative morphometry, spatial transcriptomics, multiplex imaging, and region-resolved genomics could therefore move villous architecture from an observer-dependent category to a continuous, biologically interpretable marker of early malignant transition. Such a shift may improve adenoma classification, refine surveillance strategies, and identify high-risk lesions before conventional thresholds for advanced histology are reached.

## 1. Introduction

Conventional colorectal adenomas are the principal precursor lesions of colorectal cancer and are routinely classified by architectural features [1,2,3]. Villous morphology is among the most clinically consequential of these features: it is associated with increased malignant potential and is incorporated into histopathological reporting and several risk-stratification frameworks [4,5]. Yet, despite its clinical importance, villous architecture is still assessed mainly by visual estimation and has no widely accepted biological definition [6,7].

Several observations suggest that villous morphology is more than an anatomic pattern. Adenomas with villous regions show altered differentiation, increased dysplastic change, mucin remodeling, and enrichment of molecular features associated with progression [8,9,10]. However, the transition from tubular to villous growth cannot be explained by genetic accumulation alone, implying that epithelial state, tissue mechanics, and microenvironmental context also shape the phenotype.

Here, we review evidence that links villous architecture to epithelial plasticity, altered lineage identity, and remodeling capacity in conventional colorectal adenomas. We argue that villous morphology is best understood as a graded biological state rather than a static endpoint. This perspective has direct clinical relevance: it explains the variable prognostic performance of villous proportion, clarifies why semi-quantitative assessment is vulnerable to observer variability, and points toward quantitative morphologic and molecular markers that could refine adenoma risk assessment.

## 2. Materials and Methods

A structured literature search was performed to identify studies relevant to villous architecture in conventional colorectal adenomas. PubMed/MEDLINE, Web of Science, and Google Scholar were searched for English-language studies published from database inception to August 2026. Search terms included combinations of “colorectal adenoma”, “villous adenoma”, “tubulovillous adenoma”, “villous architecture”, “colorectal polyp”, “APC”, “KRAS”, “GNAS”, “methylation”, “CIMP”, “YAP”, “Hippo”, “epithelial plasticity”, “fetal-like”, “regenerative”, “mucin”, “immune”, “microenvironment”, and “chromosomal instability”. Primary human studies directly comparing adenoma architecture or reporting molecular, transcriptional, spatial, or clinical features relevant to villous remodeling were prioritized. Mechanistic studies in colorectal cancer, intestinal regeneration, organoids, and mouse models were included when they provided biological context for processes not yet directly resolved in human conventional adenomas. Serrated lesions were included only when necessary to distinguish shared from pathway-specific epithelial states. Reviews were used primarily to identify additional primary literature. Studies without relevance to colorectal adenoma architecture or epithelial-state remodeling were excluded. Experimental reports lacking sufficient methodological detail were also excluded. Key methodological limitations and considerations for studies contributing the main quantitative data are summarized in the Supplementary Materials.

## 3. Histological Features and Clinical Significance of Villous Architecture

Villous architecture in colorectal adenomas is striking precisely because it appears in a tissue that normally lacks villi. The healthy colon and rectum maintain a crypt-restricted architecture governed by lineage-specifying transcriptional and epigenetic programs, including CDX2- and SATB2-dependent networks [2,11,12,13,14]. Villous projections in colorectal neoplasia therefore represent pathological remodeling of colonic epithelial organization rather than physiological recapitulation of small-intestinal villi.

Normal small-intestinal villi are organized as absorptive units composed of a polarized epithelial layer over a lamina propria core containing capillaries and a central lymphatic vessel. By contrast, the colonic mucosa is organized around invaginated crypts, with proliferative and stem-cell populations concentrated toward the crypt base and differentiated lineages positioned toward the luminal surface [15]. The appearance of finger-like projections in a colonic adenoma therefore implies redistribution of proliferative, differentiation, and polarity programs across an axis that does not normally exist in this tissue.

Histopathological classification is based on the proportion of villous architecture observed by light microscopy. Under World Health Organization criteria, lesions are classified as tubular adenomas (TAs) when the villous component is less than 25%, tubulovillous adenomas (TVAs) when it comprises 25–75%, and villous adenomas (VAs) when it exceeds 75%. Although described as villous, these neoplastic structures differ substantially from normal small-intestinal villi. A fibrovascular core is usually retained, but the epithelial compartment is less differentiated and enriched for dysplastic, immature cells rather than mature polarized absorptive and goblet-cell populations [2,16,17,18].

The emergence of villous architecture, particularly in TVAs, is accompanied by broader epithelial alterations associated with colorectal tumor progression, including increased proliferation, metabolic reprogramming, cellular plasticity, and migratory potential, together with a more dysplastic histological phenotype characterized by increased p53 immunoreactivity, a higher nuclear-to-cytoplasmic ratio, epithelial crowding, nuclear stratification, and reduced apoptosis [8,9,18,19,20,21,22]. Not all of these morphological parameters clearly distinguish between villous and tubular areas; for example, reported differences in mitotic activity between villous and tubular compartments are not fully consistent [23,24]. Villous morphology therefore correlates with, but does not fully define, the underlying biology of progression.

Mucin remodeling is another recurrent feature of villous-containing adenomas. Increased expression of intestinal and gastric-type mucins, including MUC2 and MUC5AC, has been reported, although compared with serrated lesions this pattern is more heterogeneous and usually less extensive [10,25]. Gastric-type mucins, particularly MUC5AC and MUC6, are preferentially expressed in villous regions compared with tubular areas and normal mucosa [10,26,27,28]. The association is not strictly linear: mucin reprogramming appears most evident during active remodeling and may become less conspicuous in highly dysplastic or more advanced regions [26,27,28]. This epithelial mucin-expression pattern should not be equated with the extracellular mucin accumulation that defines mucinous colorectal carcinoma. These findings support a graded and spatially organized process of lineage disturbance rather than a simple binary differentiation switch.

The clinical signal is clear, even if the biology remains imprecise. Villous and tubulovillous adenomas carry higher malignant risk than purely tubular lesions, and increasing villous proportion correlates with colorectal cancer risk in cohort and pathology studies [1,4,5,29,30,31]. Yet its independent prognostic value weakens in some analyses after adjustment for other clinicopathological variables [32]. Rather than diminishing the importance of villous morphology, this inconsistency suggests that it functions as a composite readout of proliferation, dysplasia, epithelial-state change, and microenvironmental remodeling, rather than as a single causal variable.

In routine practice, risk assessment still depends heavily on estimating villous proportion, a semi-quantitative and observer-dependent method with substantial interobserver variability [6,7]. This is especially problematic in lesions with focal or early villous change, where classification may depend on section orientation and on how observers distinguish elongated glands from true fibrovascular projections. Computational morphometry and interpretable machine-learning approaches may help by quantifying epithelial curvature, glandular orientation, branching, surface folding, and fibrovascular-core continuity [33,34,35,36]. Such measurements could convert villous architecture from a categorical estimate into a continuous phenotype and reveal biologically meaningful remodeling before conventional thresholds are reached.

**Figure 1:**
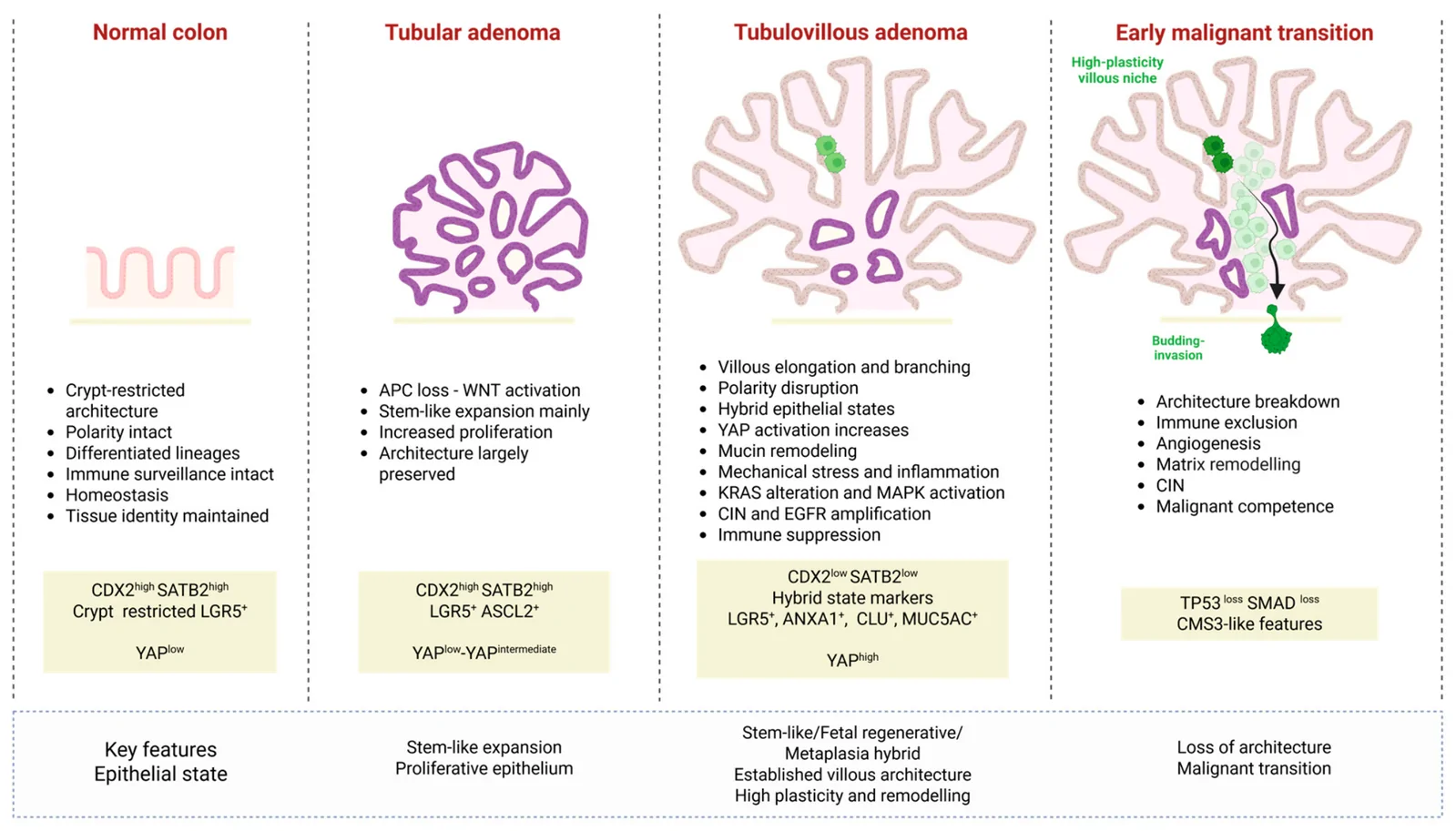
Proposed epithelial state evolution during villous progression in conventional colorectal adenomas. Schematic model showing the transition from normal crypt-restricted colonic epithelium to tubular adenoma, tubulovillous adenoma, and early malignant transformation. APC loss and WNT activation initiate stem-like epithelial expansion in tubular adenomas. During progression to tubulovillous adenoma, increased YAP activity, epithelial plasticity, mucin remodeling, KRAS/MAPK signaling, stromal remodeling, and immune attenuation accompany villous elongation and architectural instability. Localized high-plasticity villous niches may further acquire invasive competence, contributing to early malignant transition. The most likely and best-supported APC-first sequence is visualized here. However, this framework should not be interpreted as an obligatory linear progression. Different starting genotypes and varying combinations of molecular, epithelial-state, stromal, and mechanical alterations may converge on a similar villous phenotype with malignant potential, as discussed in Section 8, “The Villous State in Conventional Adenomas: Evolutionary Routes”.

The following sections examine the molecular and structural mechanisms that may generate this continuous villous phenotype and link it to premalignant progression (Figure 1).

## 4. Genetic and Epigenetic Determinants of Villous Architecture

No single genetic event appears to determine villous architecture. APC alteration is the canonical initiating event in conventional adenoma formation, but its relationship to villous remodeling is better viewed as permissive than deterministic. APC mutations frequently occur within the mutation cluster region and lead to constitutive WNT/β-catenin activity, yet pathway output depends on the retained β-catenin regulatory repeats and the nature of the second APC hit. Some studies report a higher prevalence of APC alterations in tubulovillous and villous adenomas, but this association is inconsistent, and villous architecture can arise without a detectable APC mutation [3,37,38,39]. The presence of APC loss across adenomas with widely different architectures therefore argues that WNT activation alone does not specify the villous phenotype. Architecture-stratified molecular frequencies, cohort sizes, and interpretations are summarized in Table 1, with detailed study-level information and limitations provided in Table S1.

More broadly, genetic progression and architectural remodeling do not appear to be tightly coupled. APC-deficient crypts can undergo early clonal expansion while largely preserving glandular organization, and substantial evolutionary diversification may occur before overt morphological change [40,41,42]. APC loss also perturbs epithelial organization in subtler ways, including altered daughter-cell positioning and disturbed asymmetric division or polarity, changes that may precede visible architectural distortion [43]. Accordingly, APC/WNT activation is best viewed as establishing a stem-like, growth-permissive background rather than directly specifying villous morphology.

**Figure 2:**
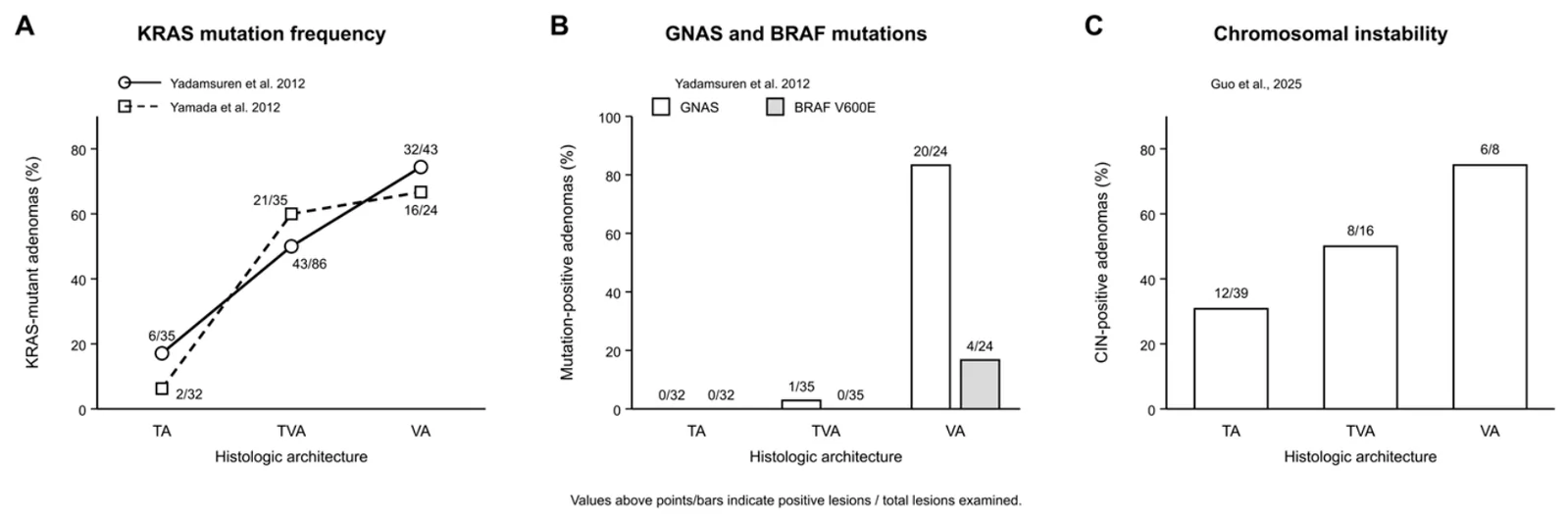
Published architecture-stratified molecular alterations in colorectal adenomas. (**A**) KRAS mutation frequencies across TAs, TVAs, and VAs in two independent cohorts [44,46]. (**B**) GNAS and BRAF mutation frequencies across adenoma architectures in the cohort of Yamada et al. [46]. (**C**) Chromosomal instability (CIN) across TA, TVA, and VA in the cohort of Guo et al. [76]. Values indicate the number of positive lesions over the total number examined. Data were extracted from the cited primary studies and were not pooled across cohorts.

Activating KRAS mutations represent one of the most frequently reported molecular alterations associated with villous architecture. KRAS mutations are enriched in tubulovillous and villous adenomas and correlate with increasing architectural complexity, greater villous proportion, and higher grades of dysplasia [8,44,45]. Stratified analyses further demonstrate a stepwise increase in mutation prevalence as villous components expand, with KRAS alterations already detectable in lesions with minimal villosity [3,9,45,46,47,48]. For example, Yadamsuren et al. reported KRAS mutations in 6/35 (17%) tubular adenomas, 43/86 (50%) tubulovillous adenomas, and 32/43 (74%) villous adenomas [44], while an independent series reported frequencies of 2/32 (6%), 21/35 (60%), and 16/24 (67%), respectively (Figure 2A) [46]. However, absolute frequencies vary substantially across cohorts, and this association has not been reproduced in every study (Table 1). Notably, mutation frequencies are often similar between tubulovillous and fully villous adenomas, suggesting that KRAS activation is linked to the emergence rather than the extent of villous architecture [3,9,45,46,47,48]. KRAS mutations can nevertheless occur in early dysplastic adenomas. In a series of 96 adenomas and in situ carcinomas, Jungwirth et al. reported KRAS mutations in 32% of low-grade adenomas, 32% of high-grade adenomas, and 45% of in situ carcinomas, with KRAS mutation significantly associated with villous histology [49]. Together with the very low KRAS frequency observed in small incident adenomas [45], these findings support a progression-associated and permissive role rather than an obligatory initiating or villous-specifying event.

Studies of histologically normal colorectal epithelium further complicate a simple APC-first sequence. Cancer-driver mutations, including KRAS, can expand clonally in normal crypts, and mutant KRAS may alter the fixation landscape for subsequent APC or CTNNB1 events [50,51,52,53]. Most conventional adenomas probably still follow an APC-first route, but these findings support alternative mutation orders that can converge on a tubulovillous phenotype.

Other alterations may contribute to the same architectural endpoint. Activating GNAS mutations show a particularly strong association with fully villous adenomas in one architecture-enriched series: mutations were detected in 20/24 (83%) villous adenomas compared with 1/35 (3%) tubulovillous adenomas and 0/32 tubular adenomas (Figure 2B) [46]. Because villous adenomas were intentionally enriched in this non-consecutive cohort, these frequencies should not be interpreted as population prevalence. BRAF V600E appears less consistent: in the same study it was absent from tubular and tubulovillous adenomas but present in 4/24 (17%) villous adenomas, whereas other conventional-adenoma series reported no BRAF mutations [46,54,55,56,57]. Thus, BRAF activation does not appear to represent a reproducible defining feature of conventional villous remodeling and remains much more strongly associated with serrated neoplasia. In some series, GNAS alterations coexist with either KRAS or BRAF, suggesting cooperation with MAPK signaling rather than an independent pathway. Villous adenomas also show broader mutational complexity, with recurrent alterations in genes such as TTN, MUC16, and GATA3 and pathway-level involvement of WNT, MAPK, PI3K, and chromatin remodeling [58,59]. The main limitation is spatial resolution: most studies rely on bulk tissue and cannot determine whether specific alterations localize to villous or tubular compartments within the same lesion. Region-resolved sequencing will therefore be essential to distinguish alterations associated with lesion size from those directly linked to local architectural remodeling.

Epigenetic data support a similar permissive and reinforcing rather than deterministic model. Genome-wide methylation studies distinguish tubular from villous-containing adenomas at the level of global methylation structure, with differentially methylated regions involving genes linked to proliferation, cell-fate regulation, stem-cell activity, and tissue remodeling [60,61]. However, these studies do not identify a single recurring methylation signature that robustly defines villous morphology across cohorts. Biological heterogeneity, bulk profiling of mixed architectural regions, and variability in pathological classification probably all contribute to this inconsistency. The most defensible interpretation is therefore that epigenetic alterations provide a modulatory background that facilitates epithelial-state transitions and signaling reinforcement rather than serving as an autonomous villous program.

MGMT is the most extensively studied example. Increased MGMT promoter methylation and reduced protein expression have been reported in adenomas with villous components, although not in every cohort [8,48,57,62]. In one architecture-focused series, MGMT promoter methylation was detected in 11/30 (37%) tubular adenomas compared with 27/31 (87%) villous-containing adenomas (*p* < 0.01) [57], whereas another cohort did not show a simple monotonic relationship between MGMT methylation and villous architecture (Table 1 and Table S1) [48]. Because MGMT loss biases O6-methylguanine repair failure toward G>A and C>T-like mutational patterns, it may increase the likelihood of specific driver events, including KRAS substitutions [59,63,64,65]. MGMT methylation is therefore better viewed as a mutationally permissive factor than as a direct morphological determinant.

The same principle applies to CIMP-related changes. Interpretation is complicated because adenoma studies have used different marker panels and thresholds. Adenomas with villous components often show increased CpG-island methylation, but this does not reproduce the canonical CIMP-high state of the serrated pathway; low or intermediate methylation appears more common than a discrete high-CIMP phenotype [57,66,67,68,69]. For example, Kakar et al. reported CIMP positivity in 8/30 (27%) tubular adenomas and 14/32 (44%) villous-containing adenomas, a difference that did not reach statistical significance (*p* = 0.08), whereas MGMT and RASSF2 methylation were significantly enriched in the same villous-containing group (Table 1) [57]. Promoter methylation of RASSF2 may be particularly relevant because RASSF2 links Ras signaling to MST/Hippo regulation [57,70,71,72,73,74]. Its silencing could reinforce proliferation, polarity disturbance, and epithelial remodeling by releasing Ras and YAP/TAZ constraints. Broader methylation changes involving WNT antagonists and lineage regulators are shared across conventional adenoma subtypes and do not uniquely identify villous lesions [75]. Overall, the genetic and epigenetic data support a state-level outcome generated by cumulative and interacting alterations rather than by any single mutation or methylation event. Paired tubular and villous regions from the same lesion will be especially valuable for distinguishing local drivers of architecture from lesion-wide passenger changes.

**Table 1:** Molecular alterations associated with villous architecture in conventional colorectal adenomas.

Molecular Feature	Representative Study	Cohort and Assay	Tubular Adenoma	Tubulovillous Adenoma	Villous Adenoma	Statistical Evidence	Main Interpretation
APC mutation	De Benedetti et al., 1994 [37]	58 evaluable sporadic adenomas; SSCP analysis of APC coding regions	15/45 (33%)	10/13 (77%) †	—	OR 6.67; *p* = 0.005; association retained after multivariable adjustment	Detectable APC mutations were enriched in adenomas containing a villous component, although this association has not been uniformly reproduced across studies.
APC mutation	Mulkens et al., 1998 [39]	32 sporadic adenomas; PCR–TGGE screening of the APC mutation cluster region	36%	71% †	—	NS for histology; *p* < 0.05 for adenoma size	Showed the same directional association with villous histology, but the small cohort and restriction to the APC mutation cluster region limit interpretation.
KRAS mutation	Maltzman et al., 2001 [47]	738 adenomas; direct sequencing of codons 12/13	48/454 (10.6%)	78/282 (27.7%) †	26% (VA *n* = 42)	OR 3.2 (95% CI 2.1–4.9); adjusted OR 2.3 (1.5–3.7)	Large cohort demonstrating an independent association between KRAS mutation and villous-containing histology.
KRAS mutation	Barry et al., 2006 [45]	303 incident adenomas after a recent clearing colonoscopy; dHPLC followed by sequencing	2/259 (0.8%)	7/44 (16%) †	—	RR 20.6 (95% CI 4.4–96.0); GEE RR 10.6 (2.7–41.9)	KRAS mutation was rare in small incident adenomas overall but strongly enriched in lesions with villous architecture, supporting a progression-associated rather than obligatory initiating role.
KRAS mutation	Yadamsuren et al., 2012 [44]	164 sporadic polypoid adenomas; PCR-RFLP of codons 12/13	6/35 (17%)	43/86 (50%)	32/43 (74%)	*p* < 0.0001	Demonstrated a strong stepwise increase in KRAS mutation frequency with increasing villous architecture.
KRAS mutation	Kakar et al., 2008 [57]	62 conventional adenomas; direct sequencing	3/30 (10%)	3/32 (9%) †	—	*p* = 0.4	Provides evidence that the KRAS–villosity association is not universal.
GNAS mutation	Yamada et al., 2012 [46]	91 conventional adenomas; direct sequencing of GNAS exon 8	0/32 (0%)	1/35 (3%)	20/24 (83%)	TA vs. VA *p* = 1.4 × 10^−11^; TVA vs. VA *p* = 7.2 × 10^−11^	Activating GNAS mutation showed marked enrichment in fully villous adenomas in this selected cohort.
BRAF V600E	Yamada et al., 2012 [46]	91 conventional adenomas; direct sequencing of BRAF exon 15	0/32 (0%)	0/35 (0%)	4/24 (17%)	Significant for VA vs. TA/TVA	BRAF was absent from TA/TVA but present in a minority of fully villous adenomas; in the same study, BRAF was substantially more frequent in serrated lesions.
MGMT promoter methylation	Kakar et al., 2008 [57]	Methylation-specific PCR	11/30 (37%)	27/31 (87%) †	—	*p* < 0.01	Strong enrichment of MGMT methylation in villous-containing adenomas.
RASSF2 promoter methylation	Kakar et al., 2008 [57]	Methylation-specific PCR	21/30 (70%)	30/32 (94%) †	—	*p* = 0.02	RASSF2 methylation increased in adenomas with villous architecture, although a direct morphogenetic role remains unproven.
CIMP-positive phenotype	Kakar et al., 2008 [57]	CIMP defined as methylation at ≥3 of 7 assayed loci	8/30 (27%)	14/32 (44%) †	—	*p* = 0.08	CIMP positivity was numerically enriched but not statistically significant, arguing against a uniform canonical CIMP-high villous state.
Chromosomal instability (CIN)	Guo et al., 2025 [76]	63 histologically classified adenomas; LC-WGS/UCAD	12/39 (31%)	8/16 (50%)	6/8 (75%)	*p* = 0.049 across histological groups	Suggests increasing CIN with architectural complexity, but evidence derives from a single small cohort, particularly for VA (*n* = 8).

Notes: Representative primary studies reporting architecture-stratified molecular data are summarized. Frequencies are shown as n/N (%) where exact numerators and denominators were explicitly available in the original publication. Data were not pooled because of substantial heterogeneity in cohort composition, adenoma size and grade, histological definitions, molecular assays, and study design. The table is intended to illustrate both reproducible associations and inter-study heterogeneity rather than provide pooled prevalence estimates. † Tubulovillous and villous adenomas were combined in the original analysis.

## 5. Transcriptional Organization and Epithelial State Dynamics

**Figure 3:**
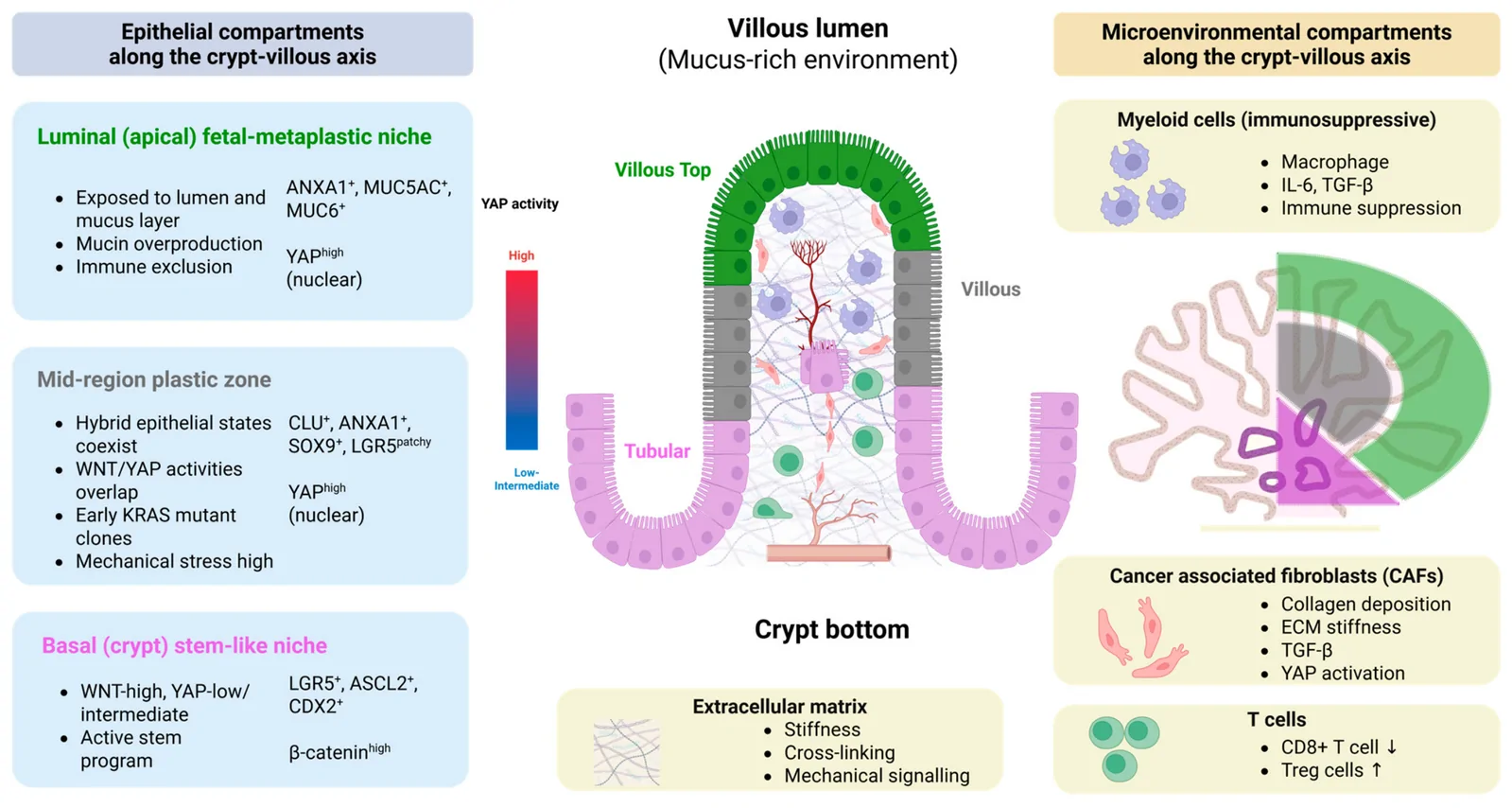
Spatial organization of epithelial and microenvironmental states along the crypt–villous axis. Schematic representation of compartmentalized epithelial states and microenvironmental remodeling in tubulovillous adenomas. Tubular/crypt-like regions can retain WNT-high stem-like features, whereas remodeling villous regions may contain regenerative and hybrid plastic epithelial states with increased YAP-associated signaling. Fetal-regenerative markers such as CLU and ANXA1 can occur within remodeling epithelial populations, while more extensively metaplastic or mucin-associated states are characterized by gastric-lineage markers such as MUC5AC and MUC6. Experimental models suggest spatial separation between crypt-bottom revival-stem-cell programs and crypt-top fetal-metaplastic programs, but an equivalent fixed top–bottom organization has not yet been established across human conventional adenomas. These epithelial changes coexist with extracellular matrix remodeling, CAF activation, myeloid enrichment, reduced cytotoxic T-cell activity, and immune compartmentalization. Upward and downward arrows indicate relative increases and decreases in cell abundance, respectively; the blue-to-red color scale indicates low-to-high YAP activity. The figure therefore represents a conceptual spatial framework rather than an obligatory compartmental organization.

Spatial and high-resolution transcriptional studies suggest that villous adenomas are not transcriptionally uniform. Instead, they contain organized epithelial programs in which canonical intestinal differentiation coexists with tissue-remodeling, stress-response, and fetal-like features [77]. This observation is central to the present framework because it connects architecture to cell state rather than treating morphology as a superficial endpoint (Figure 3). Because Ref. [77] is currently available as a bioRxiv preprint, this spatial framework should be considered emerging evidence pending peer-reviewed validation.

Adenomatous epithelium is consistently enriched for WNT-high stem-like programs relative to normal mucosa. Markers such as LGR5, OLFM4, ASCL2, AXIN2, RNF43, EPHB2, and SOX9 are increased in tubular and tubulovillous adenomas, and stem-cell markers are no longer restricted to the normal crypt base [3,78,79,80,81,82]. In conventional adenomas, LGR5 expression can become diffuse or patchy along dysplastic glands rather than retaining the stereotyped crypt-base distribution seen in normal mucosa, indicating expansion and spatial disorganization of the stem-cell compartment rather than simple basal enrichment [81,82]. These features are broadly consistent with proliferative epithelial programs enriched for WNT activity and aligned with iCMS2-like biology [83,84].

Alongside this stem-like program, fetal-like epithelial states have emerged as a second axis of adenoma identity [3,84]. These states can be understood within a broader framework of epithelial plasticity, in which adult intestinal epithelial cells transiently adopt alternative differentiation programs in response to injury, inflammation, or tumorigenic stress. This process, described as dedifferentiation, fetal-like reversion, or regenerative stem cell formation, has been observed across experimental and clinical settings [85,86,87]. Comparative analyses of adult and fetal intestinal epithelium have identified distinct transcriptional programs underlying these states: adult epithelial cells express canonical markers such as OLFM4, ASCL2, CDX2, GATA4, TCF7L2, SMOC2, and HOPX, whereas fetal-like states are enriched for genes including SOX17, CLU, TACSTD2 (TROP2), GJA1, ANXA1, ANXA3, and ANXA6, often accompanied by reduced CDX2 expression and partial loss of intestinal lineage identity [11,13,88]. These markers should not be interpreted as state-specific in isolation: TACSTD2/TROP2, for example, is induced in both regenerative/fetal and metaplastic contexts, whereas CLU and ANXA1 are more consistently associated with regenerative/revival programs. Reduced CDX2 expression is particularly relevant because CDX2 is not simply a differentiation marker; it actively maintains intestinal lineage identity, and its loss in adult intestinal stem cells results in stable reprogramming toward a pyloric-like identity [11,13,89]. Villous remodeling may therefore involve not only expansion of stemness but also partial relaxation of lineage constraint and coexistence of multiple epithelial states within the same lesion.

Although initially described in serrated neoplasia, such fetal-like epithelial programs have also been detected in conventional adenomas, including tubulovillous adenomas, where they may coexist with stem-like states [3,77,84,90]. The fetal-regenerative state is most consistently characterized by CLU and ANXA1, with relative suppression rather than complete loss of canonical LGR5-high identity. It resembles regenerative or revival stem-cell programs described during epithelial injury and repair [84,90]. Functionally, these cells are thought to act as a reserve population that supports epithelial repair and reconstitution of the LGR5+ stem cell pool following damage, and can expand beyond the crypt niche during regeneration [86,87]. Direct spatial evidence in human conventional adenomas remains limited, although recent spatial profiling has identified CLU+ regenerative/revival-like epithelial populations within remodeling adenoma regions [77]. A second phenotype, the fetal-metaplastic state, is characterized by expression of gastric or non-intestinal markers such as MUC5AC and appears to reflect lineage reprogramming toward alternative epithelial identities. MUC5AC is a more useful anchor for this state than TACSTD2/TROP2, which is shared with regenerative programs. Experimental work shows a clear spatial separation between crypt-bottom CLU+ revival cells and crypt-top fetal-metaplastic cells expressing ANXA10 in the small intestine or MUC5AC in the colon [90]. However, this top–bottom organization has not yet been established as a general feature of human conventional tubulovillous or villous adenomas. In human lesions, a preferential luminal or top-down distribution of metaplastic markers is better established in serrated neoplasia than in conventional adenomas [3,90]. Accordingly, a similar spatial arrangement in conventional villous remodeling should be considered a plausible model rather than a proven architectural rule.

Together, these observations place stem-like, fetal-regenerative, and fetal-metaplastic programs along a dynamic spectrum of epithelial plasticity. Evidence from colorectal cancer indicates that cells can shift between LGR5+ stem-like and fetal-like programs through intermediate states shaped by intrinsic mutations and extrinsic signals. APC loss favors stem-like programs, whereas YAP, TGF-β, IFN-γ, and MAPK signaling promote fetal-regenerative features [84]. These programs are neither fixed nor mutually exclusive, and hybrid/intermediate states are likely to occur within the same lesion.

These mechanisms should still be interpreted with caution. Much of the mechanistic detail comes from colorectal cancer, regeneration, organoids, or mouse models rather than from region-matched human conventional adenomas. The pathways described above are therefore likely contributors, not yet proven drivers, of villous remodeling. A recent bioRxiv preprint frames these transitions as oncofetal reprogramming rather than irreversible conversion between fixed cell types. Following APC loss, suppression of retinoid X receptor activity can permit YAP/AP-1-dependent transcriptional programs and create a persistent memory of fetal-like identity [91]. Although this mechanism has been defined mainly in colorectal cancer and organoid models, it offers a plausible explanation for how genetically similar epithelial cells within the same adenoma can occupy different transcriptional states.

The operational definitions, representative markers, spatial patterns, and directness of evidence for the three epithelial-state programs discussed here are summarized in Table 2.

## 6. YAP Integrates Mechanical and Oncogenic Signaling

**Figure 4:**
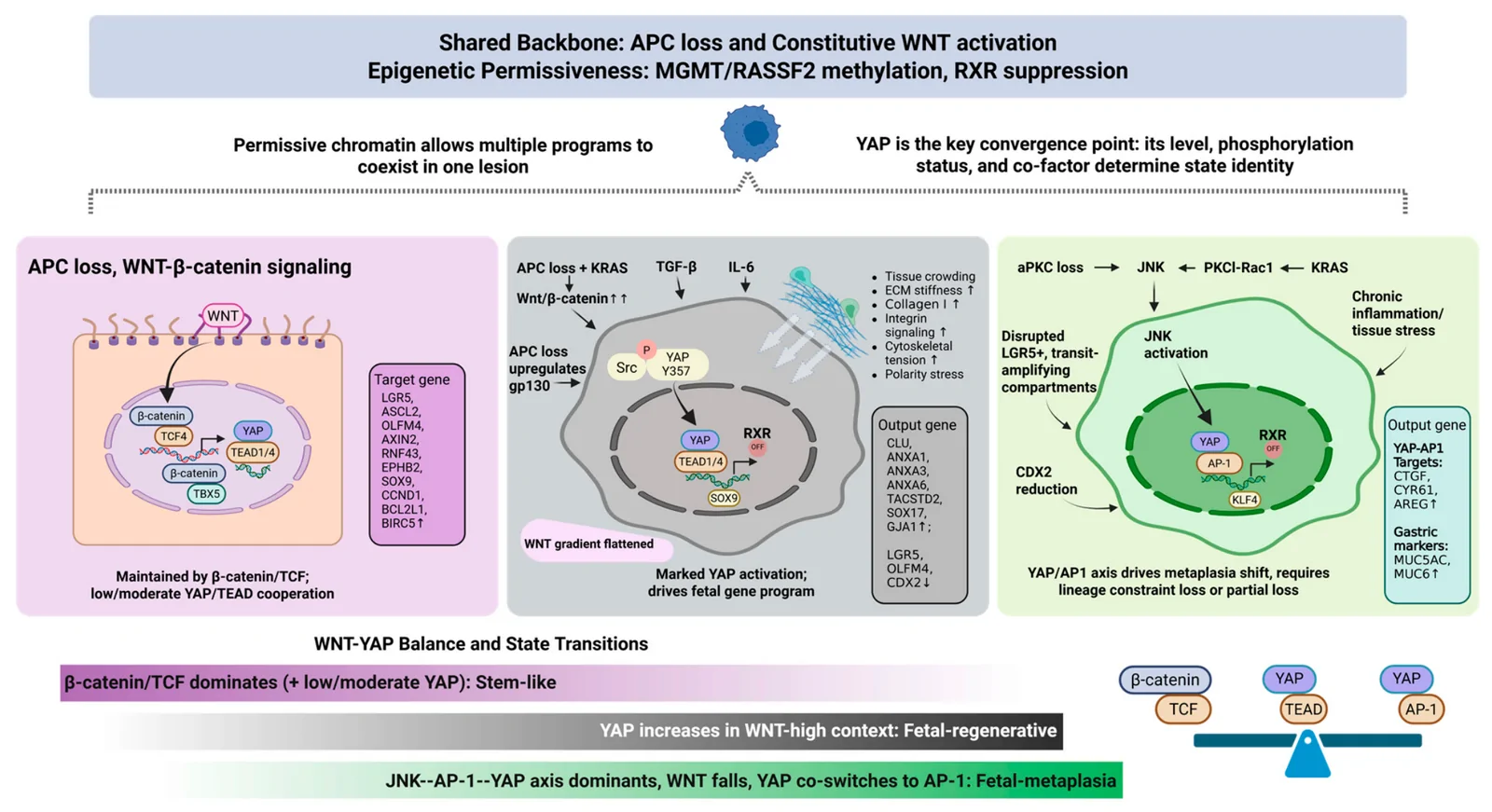
YAP-centered signaling model linking WNT activation, epithelial plasticity, and fetal-like reprogramming. Proposed mechanism by which APC loss, WNT–β-catenin activation, epigenetic permissiveness, and microenvironmental stress converge on YAP-dependent epithelial state switching. In WNT-high lesions, β-catenin/TCF activity supports stem-like programs, while increasing YAP activity promotes fetal-regenerative states through TEAD-dependent transcription. Under stronger stress or reduced lineage constraint, JNK–AP-1–YAP signaling may drive fetal-metaplastic reprogramming with gastric marker expression. Arrows indicate the proposed direction of signaling or regulatory influence. Purple, gray, and green backgrounds denote WNT/β-catenin-dominant stem-like, YAP-associated fetal-regenerative, and JNK–AP-1/YAP-associated fetal-metaplastic signaling states, respectively; the gradients indicate transitions between these configurations. These signaling configurations are conceptual and context-dependent rather than discrete, obligatory states. The balance between WNT, YAP, TEAD, and AP-1 activity may therefore shape the coexistence of stem-like, fetal-regenerative, and fetal-metaplastic states within villous lesions.

YAP1 (YAP) provides a plausible molecular bridge between tissue architecture and epithelial state (Figure 4). As a central Hippo-pathway effector, YAP integrates mechanical, architectural, inflammatory, and oncogenic cues into transcriptional programs that govern proliferation, lineage identity, and plasticity. Through this role, YAP may contribute to the ability of APC-driven lesions to access structurally complex and fetal-like epithelial states, although YAP activation itself is not specific to villous architecture.

As adenoma size increases, epithelial crowding within confined glandular structures generates compressive and tensile forces that actively feed back onto epithelial signaling. Increased tissue density and nuclear deformation promote YAP nuclear translocation. Concurrently, progressive extracellular matrix stiffening and reinforced focal adhesion signaling further sustain YAP activity, effectively coupling tissue architecture to transcriptional output [102,103,104,105,106]. Extracellular matrix remodeling provides an additional biochemical route for this activation: increased collagen I deposition and enhanced integrin signaling promote actomyosin contractility and focal adhesion dynamics, reinforcing YAP nuclear localization and supporting fetal-like transcriptional programs [97,107]. Most direct mechanistic evidence for this ECM–YAP coupling comes from regeneration models and other experimental systems rather than villous adenomas themselves, so its role in neoplastic villous morphogenesis remains inferential.

YAP also operates in close coordination with WNT–β-catenin signaling. Immunohistochemical analyses across adenoma subtypes show a strong association between phosphorylated YAP (Ser127) and nuclear β-catenin, correlating with lesion size, pathological progression, and villous architecture [80]. Cytoplasmic YAP can attenuate WNT signaling through interactions with Dishevelled proteins, whereas WNT signaling can directly induce YAP expression through β-catenin/TCF4 binding within the YAP1 locus [108,109]. In addition, WNT signaling modulates the availability of TEAD transcription factors, which are essential for YAP-mediated transcription. TEAD1 and TEAD4 are shared targets of both pathways, while TEAD2 appears more WNT-dependent and TEAD4 expression is broadly elevated in colorectal adenomas at levels comparable to SOX9 [93]. YAP and β-catenin also cooperate directly at stem cell maintenance genes such as LGR5 and CCND1 and can form higher-order complexes with TBX5 to promote anti-apoptotic gene expression [110,111].

APC loss can also activate YAP through mechanisms beyond canonical WNT signaling. APC scaffolds Hippo-pathway components such as SAV1 and LATS1 to facilitate YAP phosphorylation; APC loss therefore reduces YAP phosphorylation and promotes nuclear accumulation [112]. APC deficiency also increases epithelial sensitivity to inflammatory cues by upregulating IL6ST (gp130), enhancing responsiveness to IL-6 family cytokines and activating Src-family kinases that phosphorylate YAP at tyrosine 357, thereby stabilizing YAP and promoting nuclear localization [113,114]. These mechanisms may act sequentially: Hippo disruption produces an initial rise in nuclear YAP, while a gp130–Src feed-forward loop sustains activation. Compensatory LATS1 upregulation may partly restrain this process by promoting inhibitory serine phosphorylation and degradation.

## 7. YAP-Dependent Fetal Reprogramming

YAP-dependent state switching offers a unifying model for how stem-like, fetal-regenerative, and fetal-metaplastic programs coexist in conventional adenomas. Rather than representing fixed levels of WNT and YAP activity, these states appear to occupy context-dependent positions along a WNT–YAP plasticity spectrum. Experimental studies suggest that regenerative states can emerge within a WNT-permissive background as YAP activity increases, whereas more extensively metaplastic states can occur when adult WNT/stem-cell identity is weakened and AP-1/YAP-associated transcription becomes dominant (Figure 4) [90].

WNT signaling functions both as a driver of stem-like identity and as a permissive input for YAP-dependent transcription. WNT–β-catenin signaling promotes TEAD transcription-factor expression, enabling canonical YAP–TEAD output. Within this permissive range, epithelial identity may depend on the balance between β-catenin/TCF- and YAP–TEAD-driven transcription: WNT-dominant states maintain LGR5 and OLFM4, whereas rising YAP activity redirects cells toward regenerative targets including CLU and ANXA1. When WNT signaling falls and YAP activity remains high, YAP may instead cooperate with AP-1 to drive fetal-metaplastic programs marked by MUC5AC and other non-intestinal/gastric-lineage features. Thus, the WNT–YAP balance may determine whether cells remain stem-like, acquire fetal-regenerative features, or shift toward fetal-metaplastic identity.

Experimental studies indicate that YAP/TAZ activity can be necessary for injury-associated fetal/regenerative reprogramming and, in some settings, sufficient to induce major components of this state, but these findings should not be generalized into a universal necessary-and-sufficient rule for human adenomas [86,87,94]. During regeneration, this reprogramming is tightly restrained and epithelial cells access regenerative states when homeostatic constraints are disrupted, such as following injury or loss of stem-cell function. In regenerative models, adult ISC markers such as LGR5 and OLFM4 can be suppressed while CLU-, ANXA1-, or TACSTD2-associated programs expand. SOX9 is required for this transition, as its inactivation prevents both adenoma formation and the emergence of fetal-regenerative features while restoring multilineage differentiation. Human FAP adenoma organoids further show that SOX9 suppression reduces fetal-associated genes including TACSTD2/TROP2 and CLU and restores differentiation [92].

The spatial distribution of YAP activity further shapes this process. In the homeostatic intestinal epithelium, YAP localization and activity are spatially regulated within crypt compartments; following injury, nuclear YAP can expand more broadly through mechanisms that include Src-family kinase signaling [93,113]. Transit-amplifying and progenitor cells can then engage proliferative and regenerative programs while canonical stem-cell markers such as LGR5 and OLFM4 are relatively suppressed [93]. In APC-mutant lesions, YAP-dependent regenerative signaling becomes increasingly prominent during progression, but APC loss alone is not sufficient to impose uniform nuclear YAP activity [93]. These observations support a model in which adenomatous epithelium can access regenerative-like states beyond the normal crypt hierarchy rather than following a fixed spatial YAP gradient.

Importantly, sustained YAP activation does not uniformly enhance tumor fitness. Constitutive activation of YAP can impair re-entry into canonical stem-like states, limiting tumor growth and metastatic colonization in some models, indicating that dynamic switching between epithelial states, rather than stable fixation in a regenerative state, is required for efficient tumor progression [95,115]. Similar plasticity occurs in adenomas, where progenitor-like populations can reacquire LGR5 expression and stem-like properties under appropriate stimuli, consistent with reversible transitions between epithelial states [82].

The fetal-metaplastic phenotype appears to arise under distinct signaling conditions and remains less well defined. Recent studies implicate a JNK–AP-1–YAP signaling axis in driving this program. Loss of atypical protein kinase C (aPKC; PKCλ/ι) activates JNK signaling, depleting intestinal stem cells and inducing an AP-1/YAP-dependent metaplastic program. In inducible experimental models, loss of LGR5+/OLFM4+ stem-cell identity precedes the appearance of CLU+ revival stem cells at the crypt bottom and fetal-metaplastic cells at the crypt top, marked by ANXA10 in the small intestine and MUC5AC in the colon [90]. These findings support spatiotemporally distinct regenerative and metaplastic responses rather than a single continuous cell population. The requirement for broader epithelial disruption beyond the LGR5+ compartment also argues against a simple model in which fetal-metaplastic cells arise directly from stem cells alone [90]. Although most prominently described in serrated lesions, this JNK/AP-1/YAP axis has also been observed in a subset of conventional adenomas [3,90]. Microenvironmental factors such as chronic inflammation likely further promote fetal-metaplastic features. Furthermore, YAP can promote goblet-like differentiation programs through interactions with KLF4, providing a potential mechanistic link to mucin-rich, fetal-metaplastic epithelial phenotypes [100].

## 8. The Villous State in Conventional Adenomas: Evolutionary Routes

The villous state is unlikely to arise through a single linear route. Instead, conventional adenomas may reach a similar architectural endpoint through distinct, non-mutually exclusive trajectories that share increased plasticity, altered polarity, and tissue remodeling.

In conventional adenomas, APC loss establishes a permissive, WNT-driven stem-like background upon which additional alterations act cumulatively. Early promoter methylation of MGMT and RASSF2 is already detectable in small adenomas; MGMT silencing biases repair failure toward G>A and C>T-type mutations, including in KRAS, while RASSF2 methylation removes inhibitory constraints on RAS signaling and Hippo/MST regulation [63,64,74]. Acquisition of activating KRAS mutations within this primed background reinforces MAPK signaling and engages bidirectional crosstalk with WNT. GSK3β inhibition stabilizes both β-catenin and KRAS, while ERK-mediated LRP6 phosphorylation enhances β-catenin/TCF activity [116,117]. At the tissue level, KRAS promotes clonal expansion through increased crypt fission, enabling lateral spread of dysplastic clones [40,53,118,119,120] and disrupts epithelial polarity through MYC-dependent effects on apical–basolateral organization [121] and through a Ras–PKCι–Rac1 axis that repurposes atypical PKC from polarity maintenance toward cytoskeletal remodeling and migratory behavior [122,123].

KRAS signaling further weakens epithelial cohesion through tyrosine phosphorylation of β-catenin and its dissociation from E-cadherin [124], consistent with progressive changes in E-cadherin/β-catenin localization observed with increasing villous architecture [125], and contributes to chromosomal instability through MYC-driven mitotic errors [126]. Locally, YAP-induced amphiregulin establishes autocrine/paracrine EGFR feedback that reinforces proliferation [113,127], consistent with increased EGFR, AREG, and EREG expression in adenomatous relative to normal tissue [128,129]. EGFR copy-number gain could in principle render such lesions more self-sustaining, although this remains to be directly tested.

Cancer-driver mutations in histologically normal crypts raise the possibility of a KRAS-first route in a subset of lesions [50,51,52]. The APC-first sequence remains the best-supported model, but different starting genotypes may nevertheless converge on a similar villous phenotype.

Physical tissue forces may provide another convergent route. During normal intestinal development, mechanical forces contribute to villus formation through coordinated epithelial–mesenchymal growth and tissue folding [130,131,132,133,134,135]. Although the underlying physical mechanisms vary across developmental models, analogous mechanical instabilities could contribute to villous folding in adenomas, although this has not yet been demonstrated in neoplastic tissue. Defining which molecular trajectories generate this physical endpoint, and which stromal clusters and epithelial–mesenchymal interactions govern it, remains an important priority.

## 9. Immune Remodeling During Villous Progression

Immune profiling studies indicate that immune contexture changes alongside architectural progression in preinvasive colorectal lesions (Figure 3).

Direct architecture-stratified evidence now supports an association between increasing villous content and reduced immune activity. In a study of 101 preinvasive colorectal lesions, increasing villous percentage was associated with significantly lower densities of IL-17A, IL-6, mast cells, and NK-cell ligand expression, with a trend toward lower IFN-γ. Lesions with 76–100% villous histology also showed significantly lower mast-cell, IFN-γ, IL-17A, and IL-6 signals than lesions with minimal villous architecture [136]. More recently, multispectral immunofluorescence across 790 colorectal precursor lesions showed that greater villous component within conventional adenomas correlated with fewer intraepithelial CD3+CD8+ T cells [137]. These studies provide direct human evidence that increasing villosity is associated with attenuation of several cytotoxic and inflammatory immune features, although they do not establish immune suppression as a cause of villous morphogenesis. Meta-analysis of sporadic adenoma transcriptomes found lower enrichment of CD8+ T-cell, NK-cell, and MHC class I signatures in adenomas than in adjacent or healthy mucosa, together with a lower CD8+/Treg ratio and increased TGF-β and Th17-associated activity [138]. These findings suggest stage-dependent immune dynamics in which inflammatory and regulatory signals emerge early, whereas selected cytotoxic features are progressively attenuated during architectural and malignant progression.

Unsurprisingly, conventional adenomas are most commonly aligned with a stemness-associated epithelial phenotype resembling the intrinsic consensus molecular subtype 2 (iCMS2), characterized by relatively low cytotoxic immune infiltration and a CD4+ T cell-dominant immune response. In contrast, serrated lesions and MSI-high colorectal cancers display metaplastic epithelial programs (iCMS3-like) accompanied by more prominent CD8+ T cell-mediated immunity [3,83,139].

Changes in stromal and immune cell composition further reinforce this environment. Advanced polyps show enrichment of regulatory T cells and pre-cancer-associated fibroblasts, alongside increased myeloid infiltration and features of immune exhaustion [140]. While IL33/ST2-associated Treg signaling has been described in colorectal lesions, its association with villous morphology appears limited [141].

Epithelial-intrinsic features may also contribute to immune evasion. Mucin-rich phenotypes, which are enriched in villous histology, can suppress immune responses through glycan-mediated interactions, facilitating tumor escape and providing a plausible explanation for reduced immune infiltrate density observed in villous-containing lesions despite ongoing epithelial expansion [136].

Overall, current evidence supports immune attenuation as a reproducible correlate of increasing villous architecture, whereas causality remains uncertain. The directness and strength of evidence supporting the molecular, epithelial-state, mechanical, stromal, and immune mechanisms proposed in this review are summarized in Supplementary Table S2.

## 10. Malignant Potential of the Villous State

The clinical relevance of villous architecture ultimately rests on its association with malignant potential. Villous-containing adenomas show genomic, epigenetic, and transcriptional alterations that overlap with later stages of the adenoma–carcinoma sequence.

Evidence for increasing chromosomal instability (CIN) with villous architecture remains limited. In one recent low-coverage whole-genome sequencing series, CIN positivity increased from 12/39 (31%) tubular adenomas to 8/16 (50%) tubulovillous adenomas and 6/8 (75%) villous adenomas (*p* = 0.049; Figure 2C) [76]. These estimates derive from a single relatively small cohort and should be interpreted cautiously, particularly for the villous subgroup (*n* = 8). Recurrent copy-number alterations included gains involving 13q, chromosome 7, 8q, chromosome 20, and chromosome 6, together with losses involving 18q, 14q, 8p, chromosome 4, and chromosome 5 [76]. The same study reported frequent copy-number gains involving regions encompassing the EGFR and MYC loci. Because these alterations were inferred from low-coverage whole-genome sequencing, they are more appropriately interpreted as regional or arm-level copy-number gains rather than definitive focal gene amplifications. Additional copy-number studies indicate partially overlapping but non-identical genomic landscapes across adenoma subtypes, supporting a broadly shared genomic instability rather than a strictly linear progression model [60]. However, chromosome 7, enriched for both copy-number variation and differentially methylated regions, may be particularly relevant, since it harbors relevant mucin-associated remodeling genes [60].

Other indicators of genomic instability also increase with villous progression. In familial adenomatous polyposis samples, homologous recombination deficiency scores rise from tubular adenomas to villous adenomas and carcinomas, accompanied by alterations in CIN-associated genes (APC, RAS, SMAD4, TP53) and DNA repair genes (SUZ12, KMT2C, BCLAF1, RUNX1, ARID1B), suggesting overlapping CIN, MSI, and CIMP-related processes [142]. At the protein level, p53 accumulation appears early during architectural progression and is detectable even in adenomas with limited villous components [8].

Villous-containing adenomas can show carcinoma-associated molecular features despite low-grade histology. Multi-omics analyses have identified upregulation of genes involved in colorectal cancer progression, including CXCL5, GREM1, IGF2, CTGF, and PLAU, with enrichment of extracellular matrix organization, ECM–receptor interaction, cell-cycle regulation, DNA repair, DNA replication, p53 signaling, collagen organization, and glycosylation pathways [143]. In the same study, TP53, FBXW7, PIK3CA, KIAA1804, SMAD2, and SMAD4 mutations were preferentially associated with carcinoma-containing polyps, while ERBB3 and E2F8 alterations suggested early malignant transformation in some villous-containing adenomas [143]. Comparative sequencing further supports a continuum between tubulovillous adenomas and carcinomas, with shared APC, TTN, MUC16, and KRAS mutations and predominantly C>T transition spectra, while TP53 and SPTA1 mutations are more frequent in carcinomas and SOX9 and ARID5B mutations are enriched in adenomas [58,144].

Genome-wide hypomethylation accompanies the normal-to-adenoma-to-carcinoma transition, with differential methylation enriched on chromosome 6, including the major histocompatibility complex region, and alterations near NEU1 implicating sialylation pathways in progression and immune evasion [145].

One possible interpretation is that invasive competence emerges when a final polarity-based constraint is lost, rather than when an entirely separate genetic event occurs. Once apico-basal polarity is sufficiently compromised, proliferative and plastic epithelial cells may lose the directional cues that keep expansion crypt-restricted, enabling extension through the basement membrane. Loss or functional repurposing of aPKC, together with altered E-cadherin/β-catenin localization during villous progression, offers a plausible mechanism for this final step [90,122,124,125].

## 11. Concluding Remarks and Future Perspectives

Villous morphology has long served as a descriptive histological feature for clinical risk stratification in conventional colorectal adenomas. The evidence reviewed here supports a broader interpretation: villous architecture marks a graded, convergent epithelial state characterized by stem-like and fetal-like plasticity, altered polarity, mucin remodeling, immune attenuation, stromal remodeling, and molecular features that overlap with early malignant transformation. This state can be reached through interacting genetic, epigenetic, mechanical, and microenvironmental routes rather than through a single obligatory pathway.

This reframing has practical implications for pathology and surveillance. Current WHO classification relies on semi-quantitative estimation of villous proportion, which may miss early or focal remodeling and is vulnerable to interobserver variability. Quantitative artificial intelligence-assisted assessment of epithelial curvature, gland elongation, branching, surface folding, and fibrovascular-core organization could complement conventional pathological classification by converting villous morphology from a categorical estimate into a continuous phenotype. In principle, such measurements could improve reproducibility, identify focal high-risk architecture that is underrepresented in limited tissue sections, and provide a standardized variable for integration with dysplasia grade, lesion size, multiplicity, molecular features, and clinical history. If prospectively validated, such continuous architectural measures could also be integrated with established clinicopathological factors to refine clinical risk stratification and post-polypectomy follow-up.

## Figures and Tables

**Table 2:** Epithelial cell-state programs associated with villous remodeling in colorectal adenomas.

Epithelial State	Operational Definition and Representative Markers	Spatial Distribution/Location	Signaling and Biological Context	Evidence Directly Relevant to Conventional Adenomas	Representative References
Stem-like/crypt-base-columnar-like (CBC-like)	Adult intestinal stem-cell program characterized by LGR5, OLFM4, ASCL2, AXIN2, RNF43, EPHB2, with additional stem-associated genes including SOX9 and SMOC2. This state is distinguished from regenerative states by stronger canonical WNT/CBC identity.	In normal colon, LGR5/OLFM4-associated stem cells are concentrated at the crypt base. In conventional adenomas this organization becomes disrupted: LGR5 can be expressed broadly throughout dysplastic glands rather than remaining confined to the base. Spatially resolved TVA data also identify proliferative/stem-associated epithelial populations within adenoma tissue, although a strict crypt-base localization is no longer maintained.	Primarily associated with canonical WNT–β-catenin signaling and APC loss. APC-mutant conventional adenomas are enriched for WNT-driven stem-cell expansion. The CBC/RSC balance is plastic rather than fixed. Conventional APC-pathway neoplasia is generally biased toward the CBC-like side of this spectrum relative to RSC-biased serrated lesions.	Strong direct human evidence. Chen et al. identified adenoma-specific cells enriched for LGR5, OLFM4, ASCL2, AXIN2, RNF43 and EPHB2, consistent with WNT-dependent stem-cell expansion. Baker and Jang independently showed expansion and spatial redistribution of intestinal stem-cell markers in conventional adenomas. Gil Vazquez et al. found human tubulovillous adenomas preferentially enriched toward the CBC phenotype.	[3,81,82,84], supported by [78,79,80,83]
Fetal-regenerative/revival-stem-cell-like (RSC-like)	Injury-associated, fetal-like regenerative state characterized most consistently by CLU and ANXA1, with recurrent fetal/regenerative markers including TACSTD2/TROP2, GJA1, PLAUR, and context-dependent expression of other repair-associated genes. Compared with the CBC state, canonical adult stem-cell genes such as LGR5/OLFM4/ASCL2 are often reduced rather than uniformly absent.	There is no evidence for one fixed anatomical compartment in human conventional adenomas. Recent spatial TVA data identified a CLU+ regenerative/revival-like epithelial population within adenoma regions. Experimental regeneration studies similarly identify CLU+ revival populations that can contribute to restoration of the LGR5+ compartment.	Associated with YAP/TAZ-mediated regenerative reprogramming, ECM–integrin–FAK/Src signaling, inflammatory cues, IFN-γ, TGF-β, and MAPK signaling. YAP can transiently suppress the homeostatic WNT/CBC program while activating regenerative genes. The state is reversible and can interconvert with LGR5+ stem-like populations.	Moderate and emerging direct human adenoma evidence; strong experimental mechanistic support. Human FAP adenoma organoids show developmental/fetal reprogramming, with SOX9-dependent expression of fetal-associated genes including TACSTD2/TROP2 and CLU, together with impaired differentiation. Human precursor-lesion analysis supports variable CBC–RSC admixture, with conventional TVA generally more CBC-biased than serrated lesions. Direct spatial evidence for CLU+ regenerative/revival-like populations within TVA is currently provided mainly by recent spatial transcriptomic work.	[77,84,92], mechanistically supported by [86,87,88,91,93,94,95,96,97,98,99]
Fetal-metaplastic/gastric-like	More extensively lineage-reprogrammed fetal state characterized by MUC5AC and other gastric/non-colonic lineage features, frequently accompanied by reduced CDX2 and loss of normal intestinal identity. Depending on model and lesion type, markers can include ANXA10, AQP5, TFF2, TACSTD2/TROP2, MSLN and related fetal/gastric programs. MUC5AC is the most useful anchor marker, whereas TACSTD2/TROP2 alone is not specific because it can also occur in regenerative states.	Strongest direct spatial evidence comes from metaplastic/serrated biology: MUC5AC+ cells can arise at the luminal/crypt-top surface and subsequently extend downward. Kinoshita et al. experimentally separated crypt-bottom CLU+ revival cells from crypt-top fetal-metaplastic cells, demonstrating distinct spatial programs. In conventional adenomas, however, a reproducible top-down distribution of this state has not yet been established across cohorts; gastric/fetal-like epithelial programs appear to occur only in subsets.	Associated with stronger lineage-identity loss, JNK–AP-1–YAP signaling and reduced aPKC/polarity control. CDX2 loss can facilitate non-intestinal/gastric identity. Experimental lineage-reversion models also show that TGF-β/YAP–TAZ signaling can establish a persistent embryonic/fetal state with reduced dependence on canonical WNT.	Limited-to-moderate direct evidence in conventional adenomas; stronger evidence in serrated lesions, CRC and experimental models. Chen et al. provide very strong human evidence for MUC5AC/gastric metaplasia in serrated polyps but explicitly distinguish this from conventional adenoma stem-cell expansion. Kinoshita et al. detected revival and fetal-metaplastic programs in premalignant lesions of both serrated and conventional origin, but the clearest top-vs-bottom architecture derives from mechanistic models. Therefore, this state should be presented as a plausible component of villous remodeling rather than an established universal feature of TVA/VA.	[3,77,90], supported by [89,91,100,101]

Notes: Stem-like, fetal-regenerative, and fetal-metaplastic programs are summarized according to their operational molecular definitions, spatial distribution, and proposed signaling context. These states should not be interpreted as fixed, mutually exclusive cell types or as obligatory sequential stages. Instead, available studies support a continuum of epithelial plasticity in which adult stem-like and regenerative/fetal-like programs can coexist and interconvert. Importantly, the strength of evidence differs between states. WNT-driven stem-cell expansion is directly demonstrated in human conventional adenomas, whereas much of the mechanistic understanding of fetal-regenerative and fetal-metaplastic reprogramming derives from intestinal injury, organoid, mouse, serrated-lesion, and colorectal cancer models. Spatial assignments outside directly studied conventional adenomas should therefore be considered supportive mechanistic evidence rather than proof of a fixed architecture in human villous adenomas.

## Data Availability

No new primary data were generated for this review. Data presented in Figure 2 were extracted from the cited published studies.

## Supplementary Materials

The following supporting information can be downloaded at https://www.mdpi.com/article/10.3390/cells15181685/s1, Table S1: Study-level evidence linking molecular alterations to villous architecture in conventional colorectal adenomas. Table S2: Evidence supporting proposed mechanisms of villous remodeling in conventional colorectal adenomas.

## Abbreviations

AP-1, activator protein 1; APC, adenomatous polyposis coli; aPKC, atypical protein kinase C; CAF, cancer-associated fibroblast; CBC, crypt-base columnar cell; CIMP, CpG island methylator phenotype; CIN, chromosomal instability; CNA, copy-number alteration; CNV, copy-number variation; CRC, colorectal cancer; ECM, extracellular matrix; EGFR, epidermal growth factor receptor; FAK, focal adhesion kinase; FAP, familial adenomatous polyposis; HGD, high-grade dysplasia; IFN-γ, interferon gamma; iCMS, intrinsic consensus molecular subtype; ISC, intestinal stem cell; JNK, c-Jun N-terminal kinase; LC-WGS, low-coverage whole-genome sequencing; LGD, low-grade dysplasia; MAPK, mitogen-activated protein kinase; MHC, major histocompatibility complex; MMP, matrix metalloproteinase; MSI, microsatellite instability; MSI-H, microsatellite instability-high; MSS, microsatellite stable; NK, natural killer; PCP, planar cell polarity; RSC, regenerative/revival stem cell; SFK, Src-family kinase; TA, tubular adenoma; TAZ, transcriptional coactivator with PDZ-binding motif; TEAD, TEA domain transcription factor; TGF-β, transforming growth factor beta; Treg, regulatory T cell; TVA, tubulovillous adenoma; VA, villous adenoma; WNT, Wingless/Integrated signaling; YAP, Yes-associated protein.
